# Methylene tetrahydrofolate reductase (MTHFR) and vascular endothelial growth factor (VEGF) polymorphisms in Brazilian patients with Hepatitis C virus (HCV)-related hepatocellular carcinoma (HCC)

**DOI:** 10.6061/clinics/2021/e2881

**Published:** 2021-09-28

**Authors:** Sylene C.R. Carvalho, Luydson R.S. Vasconcelos, Leonardo da Fonseca, Rodrigo F. Carmo, Michele T. Tomitão, Dayse C.B.L. Aroucha, Leila M.M.B. Pereira, José Tadeu Stefano, Ulysses Ribeiro-Júnior, Claudia P. Oliveira, Flair J. Carrilho

**Affiliations:** I Hospital Universitario Oswaldo Cruz, Faculdade de Medicina, Universidade de Pernambuco, Recife, PE, BR.; II Instituto Aggeu Magalhaes (IAM/Fiocruz), Recife, PE, BR.; III Instituto do Figado e Transplantes de Pernambuco, Recife, PE, BR.; IV Universidade Federal Vale do Sao Francisco - UNIVASF, Petrolina, PE, BR.; V Disciplina de Cirurgia do Aparelho Digestivo, Instituto do Cancer do Estado de Sao Paulo (ICESP), Hospital das Clinicas HCFMUSP, Faculdade de Medicina, Universidade de Sao Paulo, Sao Paulo, SP, BR.; VI Laboratorio de Gastroenterologia Clinica e Experimental (LIM-07), Divisao de Gastroenterologia Clinica e Hepatologia, Hospital das Clinicas HCFMUSP, Faculdade de Medicina, Universidade de Sao Paulo, Sao Paulo, SP, BR.; VII Oncologia Clinica, Instituto do Cancer do Estado de Sao Paulo (ICESP), Hospital das Clinicas HCFMUSP, Faculdade de Medicina, Universidade de Sao Paulo, Sao Paulo, SP, BR.

**Keywords:** Polymorphisms, Methylene Tetrahydrofolate Reductase, Vascular Endothelial Growth Factor, Hepatocellular Carcinoma, Hepatitis C

## Abstract

**OBJECTIVE:**

The folate pathway is involved in hepatic carcinogenesis and angiogenesis. Polymorphisms in genes related to such processes, including methylene tetrahydrofolate reductase (*MTHFR*) and vascular endothelial growth factor (*VEGF*)] may play an important role in the development of hepatocellular carcinoma (HCC). The objective of this study was to evaluate *MTHFR* and *VEGF* polymorphisms in Brazilian patients with hepatitis C virus (HCV)-related HCC.

**METHODS:**

A total of 119 patients diagnosed with confirmed HCC and HCV were included in the study. SNP genotyping assays were performed using real-time PCR. VEGFA (rs2010963, rs3025039, and rs833061) and MTHFRC677T (rs1801133, rs1801131) polymorphisms were evaluated.

**RESULTS:**

The C alleles of *MTHFR* (rs1801131) and *VEGF* (rs2010963) were associated with protection against the development of multinodular HCC, while the T allele of *MTHFR* (rs1801133) was associated with a higher risk of multinodular presentation [*p*=0.04 OR 1.835 CI (1.022-3.297)]. Multivariate analysis revealed that the GG/GC genotypes of *VEGF* rs2010963 were independently associated with multinodular tumors at diagnosis (*p*=0.013; OR 4.78 CI (1.38-16.67)].

**CONCLUSION:**

Our results suggest that these polymorphisms may increase the risk of rapid tumor progression in patients with HCV infection. This subgroup of patients with HCC and who present polymorphism is more likely to be diagnosed with multinodular disease and not be amenable to receiving curative treatments. These data must be validated in larger cohorts, and the screening intervals can be customized based on genetic history.

## INTRODUCTION

Hepatitis C virus (HCV) chronic infection is one of the leading causes of chronic liver disease worldwide (1). It is estimated that 20% of the chronically infected patients will develop liver cirrhosis within 13 to 23 years, and among these, 1-4% will develop hepatocellular carcinoma (HCC) each year (2).

All etiologies leading to cirrhosis may be further complicated by HCC development, but the risk is higher in patients with viral hepatitis. One-third of the cirrhotic patients develop HCC in their lifetime (1). In Brazil, HCC has been associated with liver cirrhosis in 98% of the cases, and HCV coexists in 54% of these cases. Other etiologies of HCC-associated liver disease include hepatitis B virus (HBV) (16% of the cases), alcohol consumption (14%), non-alcoholic fatty liver disease (3%), and hemochromatosis (1%). Other etiologies include autoimmune hepatitis and primary biliary cirrhosis (4%) (3,4).

Several hallmarks of carcinogenesis remain unclear, although there is solid evidence for the role of vascular endothelial growth factor (VEGF) signaling (5). Angiogenesis is an important step in cancer development and is necessary for primary tumor cell growth, invasion, and metastasis (6). Polymorphisms in the regulatory regions of *VEGF* are associated with the occurrence of HCC and have been suggested as biomarkers of HCV-related chronic liver disease. Another protein potentially associated with HCC development is methylene tetrahydrofolate reductase (MTHFR), which plays an important role in the folate pathway; MTHFR is also involved in the maintenance of new cells and in DNA methylation, synthesis, and repair (7). Prior studies have evaluated the association between *MTHFR* polymorphisms and HCC, but the results remain controversial (8,9).

The prognosis of HCC is closely related to the stage at diagnosis. Patients with single tumors who are treated by resection or ablation present long-term survival, as do patients satisfying the Milan criteria who underwent liver transplantation. In contrast, patients with multinodular spread or extrahepatic disease have a poor prognosis despite the recent advances in intra-arterial and systemic treatments (10). Therefore, the search for biomarkers that can help identify more aggressive tumor behavior and early spread is of utmost importance.

In the present study, we aimed to verify the association of *MTHFR* and *VEGF* polymorphisms with tumor burden at diagnosis and clinical outcomes in patients with HCV-related HCC.

## METHODS

### Population and study design

This study included 119 patients with chronic HCV infection and HCC who were recruited between January 2014 and December 2016. Patient follow-up was conducted at the hepatology unit of the Oswaldo Cruz University Hospital of the University of Pernambuco and at the Institute of Liver and Transplants of Pernambuco, Northeast Region, Brazil. All patients were over 18 years of age, and chronic HCV infection was confirmed by polymerase chain reaction (PCR) (HCV-RNA+). HCC was diagnosed by computed tomography (CT) and/or nuclear magnetic resonance imaging (MRI). The radiological criteria used for HCC diagnosis were the presence of intense contrast enhancement in the arterial phase and washout of contrast in the venous-delayed phases, according to the American Association for the Study of Liver Diseases (AASLD) (11). Tumor staging was performed using the Barcelona Clinic Liver Cancer staging system (12). The exclusion criteria were human immunodeficiency virus (HIV) and/or HBV coinfection and cirrhosis of any NON-HCV etiology. To characterize the frequency of genotypes in our population, we used blood bank material from 128 individuals with normal liver enzymes (alanine and aspartate aminotransferase) and negative results for serological tests for HIV, HCV, HBV human T-lymphotropic virus (HTLV), Chagas disease, and syphilis.

### Ethics approval and consent to participate

This study was conducted in accordance with the principles of the Declaration of Helsinki. The study protocols were approved by the Committee of the Clinics Hospital of the University of São Paulo School of Medicine (No. 349/14). Informed consent was obtained from all participants included in the study.

### Blood collection and DNA extraction

Peripheral blood samples (10 mL) were collected by peripheral vein puncture in a tube containing ethylenediaminetetraacetic acid (EDTA). Genomic DNA was isolated using the PureLink™ Genomic DNA Mini Kit (Invitrogen-Life Technologies, Carlsbad, CA, USA) according to the manufacturer's instructions. DNA concentrations were quantified using a NanoDrop™ ND-1000 spectrophotometer (NanoDrop Technologies, Inc. Wilmington, USA); the following formula was used: 1 OD_260_=50 µg/mL DNA. DNA purity was assessed based on the ratio of absorbance at 260 and 280 nm, and only DNA samples with a ratio between 1.8 and 2.0 were used in this study.

### Genotyping *VEGF* and *MTHFR* single nucleotide polymorphisms (SNPs)

Genotyping was performed using the TaqMan^®^ SNP Genotyping Assays technique (Thermo Fisher Scientific**,** CA, USA) through base discrimination using fluorophore-labeled probes, with real-time PCR amplification. Polymorphisms were detected using the following assays: *VEGFA* -634G>C (rs2010963) assay (C_8311614_10) *VEGFA* 936C>T (rs3025039) assay (C_16198794_10) *VEGFA* -460C>T (rs833061) assay (C_1647381_10), *MTHFR*C677T (rs1801133) assay (C_1202883_20), and *MTHFR* A1298C (rs1801131) assay (C_ 850486_20).

Real-time PCR was performed on a Real Time PCR Step One Plus system (Applied Biosystems, Life Technologies, Foster City, USA) with TaqMan Genotyping 20× master mix (Thermo Fisher Scientific, CA, USA).

### Statistical analysis

The data were statistically analyzed using SPSS v.17 software. Allele frequencies were estimated by the gene counting method using pLINK. The chi-square test was used to determine if the genotypic distribution was in accordance with the Hardy-Weinberg equilibrium. Associations between categorical variables were evaluated using Pearson's chi-square and Fisher's exact tests, as appropriate. For the quantitative variables, the *t*-test or Mann-Whitney test was used to determine whether the variables followed a normal distribution. Differences were considered significant at *p*<0.05, and Bonferroni correction for multiple associations was used to confirm the association in a univariate model. The magnitude of these associations was estimated as odds ratios (ORs) using 95% confidence intervals (CIs). The ORs were adjusted for possible confounding variables using multivariate logistic regression.

## RESULTS

### Clinical and laboratory characteristics

A total of 119 patients with chronic HCV infection and HCC were evaluated and followed up at the hepatology unit of Oswaldo Cruz University Hospital of the University of Pernambuco and at the Institute of Liver and Transplants of Pernambuco. Among the individuals with HCC, 70.59% were men and 29.41% were women. The mean age of the participants was 62.3 years (range: 46-86 years) and the mean body mass index (BMI) was 25.6 kg/m^2^ (range: 15.2-38.5). Type 2 diabetes mellitus (T2DM) occurred in 33.61% of the patients.

Based on the Child-Pugh classification, the patients were found to be distributed in three groups (A, 60.9%; B, 36.5%; C, 2.6%). Tumor size was assessed based on the largest diameter of the largest lesion. The mean tumor size was 4.4 cm (range: 0.5-13.8 cm). Unifocal disease was observed in 46.21% of the patients, and multinodular disease was observed in 52.10% of the patients; in 2 (1.68%) patients, the number of lesions was not characterized.

The frequency of genotype 1 was found to be 67%. There were no significant differences in age, sex, BMI, and DM or in laboratory variables (total bilirubin (TB), aspartate aminotransferase (AST), alanine aminotransferase (ALT), gamma glutamyltransferase (GGT), alkaline phosphatase (ALP), platelets, and HCV genotype 1 across different Barcelona Clinic Liver Cancer (BCLC) stages. Significant variables included tumor size (*p*<0.0001), uninodular *versus* multinodular presentation (*p*<0.0001), and alpha 1 fetoprotein (AFP) levels (*p*=0.002) (Table 1).

### Analysis of genotype frequency and distribution of SNPs in *MTHFR* and *VEGF* in patients with HCC and HCV and in control subjects

The chromosomal positions of the *MTHFR* and *VEGF* polymorphisms, expected and observed frequencies, and information on Hardy-Weinberg equilibrium, minor allele, and major allele frequencies are described in Table 2. In the present study, the observed and expected genotype frequencies of the *MTHFR* and *VEGF* SNPs were consistent with the Hardy-Weinberg equilibrium (*p*>0.05).

### Association between *MTHFR* and *VEGF* and tumor characteristics

When analyzing the relationship between SNPs and the number of tumors, we observed that the *MTHFR* and *VEGF* were correlated with the presentation of the tumors as uninodular or multinodular. The *MTHFR* C (rs1801131) and *VEGF* C (rs2010963) alleles were inversely associated with multinodular HCC at diagnosis. The *MTHFR* C allele frequency (rs1801131) was 0.18 *vs* 0.32 for patients with multinodular and uninodular HCC, respectively [*p*=0.012 OR 0.46 CI (0.25-0.85)]. The *VEGF* C allele frequency (rs2010963) was 0.27 *vs* 0.43 [*p*=0.009 OR 0.48 CI (0.28-0.84)] for patients with multinodular and uninodular HCC, respectively. In contrast, the *MTHFR* T allele (rs1801133) was associated with multinodular HCC at diagnosis, with an allele frequency of 0.33 for patients with multinodular tumors and 0.21 for patients with a uninodular tumor [*p*=0.040 OR 1.83 CI (1.02-3.29)]. After Bonferroni correction for multiple associations, only one *VEGF* allele (rs2010963) was significantly associated with unimodular tumors at diagnosis (Table 3).

Investigation of an association between the *MTHFR* polymorphism and presentation of uninodular and multinodular tumors revealed a significant association for *MTHFR* rs1881131 in the genotypic and dominant models [*p*=0.036 and *p*=0.042, OR 2.18 CI (1.04-4.59), respectively]. For this SNP, the AA genotype was associated with the development of multinodular HCC. For the *MTHFR* rs1801133, the dominant genetic model exhibited a significant association, where the CT/TT genotype was associated with the development of multinodular HCC [*p*=0.04 OR 2.13 CI (1.01-4.54)]. With respect to *VEGF* rs2010963, a significant association was observed in the recessive and genotypic models, and the GG/CG genotypes were associated with the development of multinodular HCC [*p*=0.018 and *p*=0.005, OR 4.95 (CI 1.52-16.13), respectively) (Table 4).

We also evaluated the clinical and laboratory characteristics of the *MTHFR* (rs18011131 and rs1801133) and *VEGF* (rs2010963) tumors that showed significant differences in the BCLC classifications shown in Table 1, namely mean serum AFP level, Child-Pugh score, mean tumor size, and the relationship to the best genetic model (Table 5). However, no significant differences were observed in any of the aforementioned variables (Table 5).

In the binary logistic regression analysis, after adjusting for sex and age, we observed that the GG/GC genotypes for *VEGF* rs2010963 were an independent risk factor for multinodular HCC at presentation [*p*=0.013, OR 4.78 CI (1.38-16.67)] (Table 6).

## DISCUSSION

To our knowledge, this is the first study to analyze tumor characteristics in HCV-infected HCC patients and their association with *MTHFR* and *VEGF* polymorphisms, thereby revealing the relationship between these polymorphisms and tumor presentation.

Our cohort shares a close distribution of demographics with larger studies in the Brazilian population in terms of sex (59% men) and age (mean 62.3 years) (4). We also validated the values of well-known prognostic factors, such as AFP levels, which are directly related to BCLC stages (13). More advanced BCLC stages—characterized by gradual tumor growth along with an increase in nodule number, vascular invasion, and extrahepatic disease—are associated with high AFP levels.

Angiogenesis—a fundamental process that is required for the proper functioning of organs—is involved in tissue repair and a variety of pathological processes (14). VEGF can directly stimulate the growth of new blood vessels, (15) and plays an important role in HCC angiogenesis (16). Yao et al. suggested that the serum VEGF level correlates with tumor stage and HCC aggressiveness (17). Changes in plasma VEGF levels may be influenced by *VEGF* polymorphisms, and a correlation has been observed between *VEGF* polymorphisms and HCC onset (18).

In this study, we did not find a significant association between the BCLC (B/C *vs*. A) groups. Baitello et al. evaluated a *VEGF* polymorphism (C936T-rs3025039) in 102 patients from São Paulo with an HCC diagnosis and BCLC classification, but did not find any association (19). Wu et al. showed an association of HCC in HBV patients with a *VEGF* polymorphism (rs833061) but did not analyze the correlation with the BCLC stage (18). Kong et al. evaluated 416 Korean patients with a diagnosis of HCC of different etiologies and analyzed 19 *VEGF* polymorphisms. They found that for *VEGF* rs2010963, the GG genotype was associated with increased tumor size and more advanced BCLC stage (*p*=0.025), but no association was found with the number of nodules (20).

In contrast, we observed that the GG/GC genotype of *VEGF* rs2010963 was a risk factor for multinodular HCC at diagnosis. This SNP has already been shown to affect gene expression patterns and consequently alter the synthesis of active VEGF. The CC genotype of this SNP has been found to be associated with decreased *VEGF* expression. Thus, the GG/GC genotypes are associated with increased *VEGF* expression and angiogenesis, leading to more pronounced tumorigenesis and an increase in the multinodular forms (21,22). Park et al. observed that VEGF levels progressively increased as the nodules progressed from low-grade to high-grade dysplasia and early HCC (16). Deli et al. reported detection of VEGF expression in 72 patients out of the investigated 105 HCC patients (68.6%) using immunohistochemistry (22). The expression of VEGF in HCC tissues with microscopic venous invasion is significantly higher than that in HCC tissues without microscopic venous invasion (23).

The relationship between *MTHFR* polymorphisms (rs1801133) and HCC has been well studied. A meta-analysis by Jin et al. conducted showed that an *MTHFR* polymorphism, *i.e.*, rs1801133 increases the risk of developing HCC, especially in European patients with chronic liver disease; in this meta-analysis, ten studies showed that compared to the CT genotype, the TT genotype increased the risk of developing HCC (8). Kwak et al. showed no association between *MTHFR* polymorphisms, *i.e.*, rs1801133 and rs1801131 and the risk of developing HCC (24).

We analyzed the association of *MTHFR* SNPs, *i.e.*, rs1881131 and rs1801133 with all clinical variables in patients with HCC and HCV. We identified a significant association of these SNPs with tumor presentation (uninodular *vs*. multinodular) in the univariate model. The AA genotype of rs1801131 and the CT/TT genotype of rs1801133 were risk factors for the multinodular form. Functional studies on *MTHFR* variants have shown that the variant rs1801133-TT is related to decreased activity of the enzyme responsible for DNA repair, and consequently, to the onset of HCC (24-26). Additionally, a meta-analysis showed that the CC genotype of rs1881131 is a protective factor in patients with HCC (27) and other cancers (28).

Our results suggest that these polymorphisms may increase the risk of rapid tumor progression in HCC patients with HCV infection. This subgroup of patients is more likely to be diagnosed with multinodular disease and not be amenable to receiving curative treatments. If these data are further validated in larger cohorts, screening intervals can be personalized based on the genetic background. In addition, our findings may aid the development of a risk stratification model to prioritize liver transplant recipients on waiting lists based on an increased risk of HCC progression and dropout.

Although our study was the first to analyze tumor characteristics in HCV-infected HCC patients and their association with *MTHFR* and *VEGF* polymorphisms in an ethnically mixed population, more studies are needed to confirm this hypothesis in other populations with other etiologies and stratification for tumor presentation. Further, functional studies should be performed to determine the impact of these variants on the production of active molecules. Therefore, this approach may provide new insights into the clinical management of HCC patients infected with HCV, and consequently, may aid better prediction of prognosis and development of novel therapeutic measures.

Our results must be interpreted within the limitations of cross-sectional studies, *i.e.*, small sample size and heterogeneity between studies with respect to allele frequencies and penetrance in different ethnicities, in addition to variations in age, lifestyle behaviors, and medication usage.

## CONCLUSIONS

In conclusion, we have determined that the AA genotype of *MTHFR* SNPs, *i.e.*, rs1801131, rs1801133 and the CT/TT genotypes of *VEGF* rs2010963 are associated with multinodular HCC at diagnosis. The GG/GC genotypes of *VEGF* rs2010963 were shown to be independent risk factors for the development of the multinodular form of HCC. Individuals with these genotypes may have impaired cellular DNA repair and might exhibit tumor progression.

## AUTHOR CONTRIBUTIONS

Carvalho SCR, Vasconcelos LRS, Carmo RF, Tomitão MT, Aroucha DCBL, Pereira LMMB and Stefano JT contributed to the acquisition, analysis and interpretation of experimental data. Fonseca L, Ribeiro-Júnior U, Oliveira CP and Carrilho FJ contributed to the design and critical review of the manuscript.

## Figures and Tables

**Table 1:** Clinical and laboratory characteristics of Brazilian patients with HCV-related HCC, according to the BCLC (Barcelona Clinic Liver Cancer) classification.

VARIABLES	HCV-CHC (N=119)	BCLC-0/A (N=60)	BCLC-B (N=32)	BCLC-C (N=25)	*p*-value
**Clinical**					
Age (years)	62.30±8.63	62.17±8.79	62.5±9.13	63.08±7.74	0.79
Male sex	84 (70.59%)	40 (66.67%)	23 (71.9%)	21 (84%)	0.27
BMI (kg/m^2^)	25.60±4.32	26.1±4.79	24.8±3.2	25.34±4.19	0.70
T2DM	40 (33.61%)	20 (33.32%)	9 (28.12%)	8 (32%)	0.87
Tumor size	4.4±2.6	2.8±1.2	5.9±2.7	6.15±2.96	<0.0001*
**Laboratory**					
TB [mg/dL] (min-max)	1.6 (0.4-7.2)	1.7 (0.5-7.22)	1.4 (0.5-3.7)	1.74 (0.36-5.51)	0.70
AST (U/L) (min-max)	98.9 (16- 237)	103.9 (28-237)	93.2 (31-183.9)	96 (16-225)	0.46
ALT (U/L) (min-max)	90.2 (18-249)	100.7 (18-240)	84.4 (26-208)	75.92 (18-249)	0.05
GGT (U/L) (min-max)	239 (26-1629)	232.8 (26-1133)	184.7 (41-532)	320.61 (50-1629)	0.10
ALP (U/L) (min-max)	168.4 (36-630)	151.2 (51-320)	192.3 (36-618.3)	170.74 (61-630)	0.40
Platelets (min-max)	119037 (12000-324000)	107701.7 (12000-230000)	131645.2 (46000-247000)	131260.9 (55000-324000)	0.18
AFP ng/mL (min-max)	1492.5 (1.7-60500)	660(1.8-19000)	691.9 (1.7-9604)	4468.65 (1.97-60500)	0.002*
VHC genotype 1 (%)	67%	63.32%	50%	52%	0.50

**Abbreviations:** HCV, hepatitis C virus; HCC, hepatocellular carcinoma; BCLC, Barcelona Clinic Liver Cancer; BMI, body mass index; TB, total bilirubin; AST, aspartate aminotransferase; ALT, alanine aminotransferase; GGT, gamma glutamyltransferase; ALP, alkaline phosphatase; AFP: alpha 1 fetoprotein; HCV, hepatitis C virus; T2DM, type 2 diabetes.

* Statistically significant.

**Table 2:** Distribution of SNPs in Brazilian patients with HCV-related HCC and in the control group, testing for Hardy-Weinberg equilibrium (HWE).

CHR	SNP	Gene	Group	Minor allele	Major allele	Genotypes	O(HET)	E(HET)	*p-*value *(HWE)*
1	rs1801131	MTHFR	HCC	C	A	9/45/65	0.3782	0.3893	0.8136
1	rs1801131	MTHFR	Control	C	A	10/39/79	0.3047	0.3547	0.1319
1	rs1801133	MTHFR	HCC	T	C	12/42/65	0.3529	0.4008	0.2502
1	rs1801133	MTHFR	Control	T	C	10/60/58	0.4688	0.4297	0.4103
6	rs2010963	VEGF	HCC	C	G	18/48/53	0.4034	0.4567	0.2282
6	rs2010963	VEGF	Control	C	G	18/64/46	0.5	0.4761	0.7102
6	rs3025039	VEGF	HCC	T	C	0/31/88	0.2605	0.2266	0.2138
6	rs3025039	VEGF	Control	T	C	0/37/91	0.2891	0.2473	0.07268
6	rs833061	VEGF	HCC	C	T	16/59/44	0.4958	0.4723	0.6987
6	rs833061	VEGF	Control	C	T	21/54/53	0.4219	0.4688	0.2611

**Abbreviations:** SNP, single nucleotide polymorphism; CRH, chromosome; O(HET), observed relative frequency of heterozygotes; E(HET), expected relative frequency of heterozygotes; *HWE,* Hardy-Weinberg equilibrium; MTHFR, methylenetetrahydrofolate reductase; *VEGF*, vascular endothelial growth factor.

**Table 3:** Correlation between *MTHFR* and *VEGF* SNPs and number of tumors in Brazilian patients with HCV-related HCC.

CHR	SNP	Gene	Minor allele	Multinodular	Uninodular	Major allele	*p*-value	OR	(95%)
1	rs1801131	MTHFR	C	0.1855	0.3273	A	0.01267	0.4681	(0.2561-0.8555)
1	rs1801133	MTHFR	T	0.3387	0.2182	C	0.04086	1.835	(1.022-3.297)
6	rs2010963	VEGF	C	0.2742	0.4364	G	0.009456*	0.488	(0.2828-0.842)
6	rs3025039	VEGF	T	0.1129	0.1455	C	0.4573	0.7477	(0.3468-1.612)
6	rs833061	VEGF	C	0.4032	0.3636	T	0.5344	1.182	(0.6968-2.007)

Abbreviations: CHR-chromosome; SNP, single nucleotide polymorphism;* COX2,* cyclooxygenase 2; *MTHFR*, methylenetetrahydrofolate reductase; *VEGF*, vascular endothelial growth factor; HCC, hepatocellular carcinoma; HCV, hepatitis C virus; OR, odds ratio; CI, confidence interval.

* Statistically significant after Bonferroni correction for multiple comparisons (*p*<0.01).

**Table 4:** Analysis of the correlation between *MTHFR* and *VEGF* and the number of tumors in Brazilian patients with HCV-related HCC using genetic association models.

Gene (SNP)	Multinodular (62)	Uninodular (55)	Association model	*p*-value OR (95%CI)
MTHFR				
(rs1881131)				
AA	40 (0.64)	25 (0.45)	AA *vs* AC *vs* CC	*p*=**0.036**
AC	21 (0.34)	24 (0.44)	AA/AC *vs* CC	*p*=0.050, 7.47 (0.87-64.17)
CC	1 (0.02)	6 (0.11)	AA *vs* AC/CC	*p*=**0.042, 2.18 (1.04-4.59)**
(rs1801133)				
CC	28 (0.45)	35 (0.64)	CC *vs* CT *vs* TT	*p*=0.150
CT	26 (0.42)	16 (0.29)	CC/CT *vs* TT	*p*=0.372, 0.53 (0.15-1.87)
TT	8 (0.13)	4 (0.07)	CT/TT *vs* CC	*p*=**0.04, 2.13 (1.01-4.54)**
VEGF				
(rs2010963)				
GG	32 (0.52)	21 (0.38)	GG *vs* GC *vs* CC	*p*=**0.018**
GC	26 (0.42)	20 (0.36)	GG/GC *vs* CC	*p*=**0.005, 4.95 (1.52-16.13)**
CC	4 (0.06)	14 (0.26)	GG *vs* GC/CC	*p*=0.192, 1.73 (0.82-3.61)
(rs3025039)				
CC	48 (0.77)	39 (0.71)	CC *vs* CT *vs* TT	*p*=0.525
CT	14 (0.23)	16 (0.29)	CC/CT *vs* TT	-
TT	0 (0)	0 (0)	CC *vs* CT/TT	*p*=0.525, 1.41 (0.61-3.23)
(rs833061)				
TT	21 (0.34)	22 (0.40)	TT *vs* TC *vs* CC	*p*=0.815
TC	32 (0.52)	26 (0.47)	TT/TC *vs* CC	*p*=0.999, 0.85 (0.30-2.48)
CC	9 (0.14)	7 (0.13)	TT *vs* TC/CC	*p*=0.565, 0.77 (0.36-1.63)

**Table 5:** Laboratory and tumor characteristics of Brazilian patients with HCV-related HCC according to *MTHFR (rs18011131* and *rs1801133)* and *VEGF (rs2010963)* genotypes.

VARIABLE	*MTHFR rs1801131*		*p*	*MTHFR rs1801133*		*p*	*VEGF rs2010963*		*p*
	AA (n=60)	AC+CC (n=48)		CC (n=60)	CT+TT (n=48)		CC (n=18)	CG+GG (n=99)	
AFP (ng/mL)	29.5 (1.73-60.500)	60.9 (1.80-4770)	*0.65*	47.5 (1.73-20302)	26.4 (1.97- 60.500)	*0.45*	16.7 (1.80-530)	36.7 (1.73-60.500)	*0.14*
Child-Pugh	AA (n=64)	CA+CC (n=51)		CC (n=62)	CT+TT (n=53)		CC (N=17)	CG+GG (n=98)	
A	41 (64%)	29 (57%)	*0.43*	39 (63%)	31 (58%)	*0.62*	9 (53%)	59 (60%)	*0.57*
B/C	23 (36%)	22 (43%)		23 (37%)	22 (42%)		8 (47%)	39 (40%)	
BCLC	AA (n=65)	CA+CC (N=52)		CC (N=63)	CT+TT (N=54)		CC (N=18)	CG+GG (N=99)	
A	31 (48%)	29 (56%)	*0.38*	34 (54%)	26 (48%)	*0.53*	10 (55%)	50 (50%)	*0.79*
B/C	34 (52%)	23 (44%)		29 (46%)	28 (52%)		8 (45%)	49 (50%)	
Tumor size*HCC (cm)	3.7 (1.3-13.8)	4.0 (0.5-13)	*0.96*	3.9 (0.5-9.0)	3.75 (1.3-13.8)	*0.76*	3.2 (1.6-13)	3.9 (0.5-13.8)	*0.63*

* For patients with multinodular tumors, the size of the largest tumor was considered.

**Table 6:** Logistic regression analysis to predict the occurrence of multinodular tumors in 117 Brazilian patients with HCV-related HCC.

Variables	*p*-value	OR	95% CI	
Age	0.868	1.004	0.958	1.053
Male sex	0.098	2.099	0.873	5.049
MTHFR (rs1801131-AA)	0.335	1.506	0.655	3.465
MTHFR (rs1801133-CC)	0.132	0.537	0.240	1.205
VEGF (rs2010963-GG/GC)	0.013*	4.78	1.38	16.67
